# Induction of labour in nulliparous women- quick or slow: a cohort study comparing slow-release vaginal insert with low-dose misoprostol oral tablets

**DOI:** 10.1186/s12884-020-2770-0

**Published:** 2020-02-07

**Authors:** Axelina Eriksson, Sarah Jeppesen, Lone Krebs

**Affiliations:** 1 Department of Obstetrics and Gynaecology, Holbaek Hospital, Holbaek, Denmark; 2 Department of Obstetrics and Gynaecology, Hvidovre Hospital, Kettegaard Alle 30, 2650 Hvidovre, Denmark; 3 Department of Obstetrics and Gynaecology, Naestved Hospital, Naestved, Denmark; 4 Department of Clinical Medicine, University of Copenhagen, Copenhagen, Denmark

**Keywords:** Induced, Induction, Labour, Misoprostol, Angusta, Misodel, Nulliparous

## Abstract

**Background:**

This study was undertaken with the objective of comparing efficacy and safety for two different regimens using misoprostol for induction of labour.

**Methods:**

The study was set in two different hospitals in the region of Zeeland, Denmark, and designed as a prospective cohort study. Nulliparous women with unripe cervix, eligible for vaginal delivery and medical induction of labour were included. Exclusion criteria were a previous uterine scar, suspicion of growth restriction of the fetus and prelabour rupture of membranes.

One department used 25 mcg oral misoprostol tablets and the other department used 200 mcg slow-release misoprostol vaginal insert, for induction of labour.

Primary outcomes were predefined as frequency of cesarean section, tachysystole and delivery within 24 h. Secondary outcomes were: time from induction to delivery, use of additional methods for induction, postpartum hemorrhage, anal sphincter rupture, epidural, pyrexia (rectal temperature >  38.5 °C), prolonged rupture of membranes, and use of tocolysis.

**Results:**

No significant differences in women achieving vaginal delivery was found. However, a significantly increased risk of tachysystole for the vaginal administration route was observed; 28.4% compared with 2.3%. There were no events of serious neonatal asphyxia. Half of the women induced with vaginal insert delivered within 24 h, compared with 16.8% of the women induced with oral misoprostol.

**Conclusions:**

Induction with vaginal slow-release misoprostol leads to quicker delivery with an increased risk of tachysystole but with similar perinatal outcomes and rates of cesarean sections. Low-dose oral misoprostol appears to be safe, however it leads to an increased use of secondary methods and a tendency of more intrapartum pyrexia.

**Trial registration:**

Clinicaltrials.gov ID: NCT02693587 on February 262,016.

EudraCT number 2020–000366-42 on 23 January 2020, retrospectively registered.

## Background

Labour induction is a common obstetric intervention used to bring an end to pregnancies when the benefits of giving birth at that time outweigh the risks of the induction process.

The proportion of pregnancies undergoing induction varies widely between countries, in 2017 24.0% of labours were induced in Denmark [1]. When the cervix is unripe, there is general consensus that labour should be induced with either prostaglandins or a double balloon catheter [2–4]. Misoprostol is a synthetic analogue of prostaglandin E1 which acts on the cervix and on the uterine smooth muscle, facilitating cervical dilatation and promoting uterine contractions. Misoprostol administered orally or vaginally has previously been documented to be effective in labour induction [5]. The optimal dosage and route of administration have not yet been established [6–8]. A Cochrane review published in 2014 concluded that oral misoprostol is effective in achieving vaginal birth, and suggested a dosage between 20 and 25 mcg. Given that safety is the primary concern, the evidence supports the use of oral regimens over vaginal regimens because of the lower risk of hyperstimulation [5]. However, the conclusions of the Cochrane analysis have been debated since the evidence of effectiveness/equivalence is based on studies where oral misoprostol has been used in high doses (> 25 mcg), whereas the studies that concluded an increased risk of complications used smaller doses [9, 10].

This study was undertaken with the objective of comparing the efficacy and safety of a regimen using 25 mcg per oral misoprostol vs 200 mcg vaginal insert misoprostol in a population of nulliparous women with unripe cervix.

## Methods

All nulliparous women eligible for vaginal delivery and medical induction of labour were evaluated. Inclusion criteria were defined as singleton pregnancies, cephalic presentation of the fetus, a gestational age equal to or above 37 weeks. Artificial rupture of membranes was preferred for induction in women with favourable cervical conditions based on the midwife assessments. Exclusion criteria were defined as a previous uterine scar, suspicion of growth restriction of the fetus and prelabour rupture of membranes.

The women provided informed consent for the induction of labour according to local guidelines and for collection of data for this study.

This study was a prospective cohort study localised in two different departments in the region of Zeeland in Denmark, and data was collected from November 2015 until November 2017. The demographical population in the two different departments were similar with the exception of women with gestational diabetes mellitus (GDM) who were looked after in one of the departments. The two departments had similar delivery protocols and underlying rates of caesarean section (28.0 vs 30.9.5%) and instrumental delivery (18.9 vs 16.0%) for nulliparous women with induced labour, the year before this study commenced [1]. The departments used two different regimens for induction of labour; one department used 25 mcg oral tablets of misoprostol (manufactured by Azanta) and the other department used 200 mcg slow-release vaginal insert of misoprostol (manufactured by Ferring) as their standard drug of choice. The vaginal insert was removable and released misoprostol at a controlled rate of approximately 7 mcg/h, for up to 24 hours [11]. Ferring has removed the misoprostol vaginal insert from sale in 2018.

Local guidelines were elaborated for the two different departments, defining dosage and criteria for discontinuation. The dose for oral tablets, was defined as 25 mcg every 2 h with a maximum of 8 administrations per day. The treatment was discontinued when the woman was in active labour, or after 2 days. Women were examined in the out-patient clinic prior to induction, and minimum daily consecutively. Bishop score was registered prior to induction. The Bishop Score gives points to 5 measurements of the pelvic examination; dilation, effacement of the cervix, station of the fetus, consistency of the cervix, and position of the cervix [12]. In general, the women who were offered medical induction of labour had unfavourable cervical conditions since artificial rupture of membranes was preferred if possible. Midwifes decided whether artificial rupture of membranes was possible based on their subjective assessment, and not on the Bishop score.

In the department using oral misoprostol, healthy women without hypertensive disorders and no suspicion of fetal distress (according to the inclusion and exclusion criteria) were offered an out-patient regimen. They were instructed on self-administration of the tablets. They were told to contact the hospital when regular contractions started or if they had any other symptoms or questions. They were consulted over the telephone and invited in for examination if requested.

The corresponding regime for vaginal insert was defined as 200 mcg administered in the vaginal fornix posterior, and the treatment was discontinued when the woman was in active labour, after 24 h or if tachysystole occurred in combination with CTG changes.

The women induced with vaginal misoprostol insert were hospitalized from the beginning of induction through to delivery. All women were monitored with CTG for a minimum of 20 min when the contractions commenced.

### Ethical approval

The study was performed in accordance with the guidelines of the Declaration of Helsinki and was approved by the Regional Ethics Committee (no. 50213) Regional Medicines Agency and the Danish Data Protection Agency (REG-81-2015). The study was registered at clinicaltrials.gov ID: NCT02693587 on February 26; 2016 and on EudraCT number 2020–000366-42 on January 23; 2020, retrospectively registered. In the study protocol it appeared that all women who participated in the study were asked to fill out a questionnaire abort their birth experience before they left the department. In the end of this questionnaire they were asked for permission to collect information from their medical record. If they did not fill out the questionnaire, they would be contacted by telephone and asked to answer the same questions orally including the question about collecting information from their medical record. The Regional Ethics committee approved the use of verbal consent for data collection, for patients contacted by telephone.

All patients included in this study provided informed consent (verbal or written) to access their medical records, and the researcher accessing the information was/is an authorised health care professional.

Measures of outcome was predefined prior to study commencement and uploaded in clinicaltrials.gov. Primary outcomes were defined as the frequency of cesarean section (CS), hyperstimulation (defined as tachysystole with > 5 contractions in 10 min over a period of 20 min, registered on CTG) and delivery within 24 h of induction. Safety for the neonates was registered as severe neonatal asphyxia defined as umbilical artery pH < 7.0 or if missing, an Apgar of below seven at 5 minutes.

Secondary outcomes were defined as time from induction to delivery, additional methods for induction (i.e. use of a double balloon-catheter and oxytocin stimulation), postpartum haemorrhage (exceeding 1 litre), anal sphincter rupture, epidural, intrapartum pyrexia (rectal temperature above 38.5 °C), prolonged rupture of membranes (exceeding 24 h), and use of tocolysis. Furthermore, tachysystole with category III fetal heart rate patterns (observed from induction to end of second stage of labour without oxytocin use) and instrumental delivery were measured.

Data was collected prospectively from electronic medical records and all CTG recordings were reviewed.

Reporting of the results followed the STROBE guidelines.

### Statistical analyses

Outcomes were compared between the two departments with calculation of Relative Risk (RR) with 95% confidence intervals (CI). Data was collected and processed in the software “SPSS Statistics” and *p* values were calculated by chi square test and two sample t-test. Material size was calculated by the Kelsey method with a significance level of 5% and power of 80%. A 15% difference in hyperstimulation and delivery within 24 h were chosen to be of clinical relevance. The desired sample size was calculated to include a total of 378 women.

The sample size was not reached due to a smaller number of eligible women than expected during the data collection timeline. The timeline could not be expanded since the permission for one of the medications expired and a major change in electronic patient records took place in the hospitals.

## Results

**Fig. 1:**
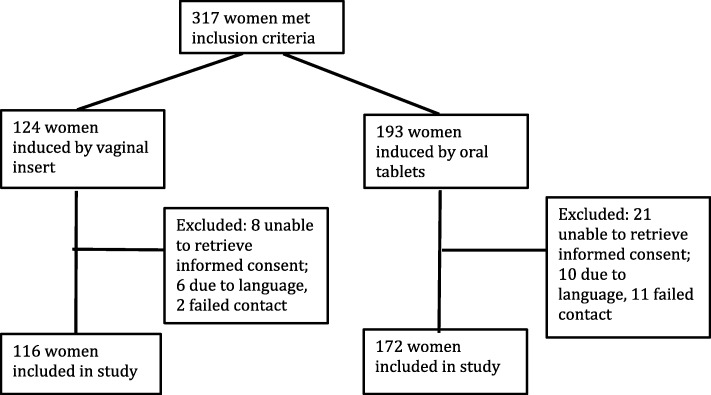
Flow chart of women in the present study

A total of 317 women met the inclusion criteria; 193 were induced with oral misoprostol and 124 women were induced with vaginal misoprostol insert. Of these, 29 women were excluded due to the women being unable to give informed consent – 16 due to language barrier and 13 were unreachable by telephone and mail (Fig. 1). Baseline demographic characteristics were similar between the groups (Table 1), except for the distribution of GDM. Medical/obstetrical indication for induction of labour included GDM and the corresponding significant difference in indications for inductions was found, as for the distribution of GDM. In a subgroup analysis excluding women with GDM the proportion of pregnancy related medical conditions was not significantly different (Additional file 1: Table S1).

**Table 1:** Patient characteristics

Induction	Vaginal insert (*n* = 116)	Oral tablets (*n* = 172)	5% *p*-value
Age (years), mean (SD)	27.8 (5.0)	27.5 (5.2)	0.62
BMI (kg/m^2^) mean (SD)	27.7 (8.4)	29.5 (6.8)	0.06
Cigarette use, n (%)	20 (17)	26 (15)	0.62
Pre-existing medical conditions, n (%)	12 (10.3)	22 (12.8)	0.53
Pre-existing psychiatrical conditions, n (%)	12 (10.3)	17 (9.9)	0.90
Pregnancy-related medical conditions, n (%)	31 (26.7)	84 (48.8)	0.002
Gestational diabetes, n (%)	1 (0.9)	37 (21.5)	< 0.001
Preeclampsia, n (%)	16 (13.8)	24 (13.9)	0.97
Hypertension, n (%)	2 (1.7)	11 (6.4)	0.06
Intrahepatic cholestasis, n (%)	3 (2.6)	5 (2.9)	0.87
Others, n (%)	9 (7.8)	7 (4.1)	0.18
Indication for induction, n (%)			
Medical/Obstetrical	45 (39)	107 (62.2)	< 0.001
Post-dates^a^	57 (49)	54 (31.4)	0.002
Other	13 (11.2)	11 (6.4)	0.15
Bishop score, mean (SD)	3.2 (1.5)	4.3 (2.1)	< 0.001
Gestational age at delivery (w + d), mean (SD)	40 + 5 (1 + 3)	40 + 5 (1 + 3)	1
Birthweight (g), mean (SD)	3636 (511)	3646 (566)	0.88

^a^Gestational age above 41 + 3

The women induced with oral misoprostol received an average of 7.2 tablets in an average time of 14.4 h (excluding an 8 h break during the night hours). Women induced with vaginal insert had it removed after 13.5 h on average, where one third of the women were unable to adhere to the regimen. The vaginal insert fell out in 19.8% of the women, 14.7% were removed due to tachysystole or hypertonic uterus with a normal CTG. In total 65.5% of the vaginal inserts were removed in adherence to the regimen.

The frequency of CS was similar in the two groups, 31.9% compared with 30.2% (Table 2). Tachysystole was reported significantly more frequently in the vaginal misoprostol insert group; 28.4% compared with 2.3% in the oral misoprostol group (RR 12.2; CI 4.5–34). Delivery within 24 h was achieved in a significantly higher proportion of the vaginal insert misoprostol group, 56% in comparison with 12.8% in the oral misoprostol group (RR 4.38; CI 2.87–6.69). Severe neonatal asphyxia was rare in both groups and did not differ significantly. There were no cases of severe asphyxia in the vaginal misoprostol group and 2 cases (1.1%) in the oral misoprostol group.

**Table 2:** Primary outcomes

Induction method	Vaginal insert *n* = 116	Oral tablets *n* = 172	RR	95%CI
Caesarean section, n (%)	37 (31.9)	52 (30.2)	1.06	0.7–1.5
Tachysystole, n (%)	33 (28.4)	4 (2.3)	12.2	4.5–34
Tachysystole with category III fetal heart rate patterns, n (%)	13 (11.2)	2 (1.1)	9.64	2.21–42
Delivery within 24 h, n (%)	65 (56.0)	22 (12.8)	4.38	2.9–6.7
Severe neonatal asphyxia^a^, n (%)	0	2 (1.1)	0.30	0.01–6.1

^a^Umbilical artery pH < 7.0 or if missing, an Apgar of below seven at five minutes

Women induced with vaginal misoprostol insert experienced a significantly shorter time from induction to delivery compared with women in oral misoprostol (Table 3). Mean time from induction to delivery for the vaginal insert was 25.6 h, 6.9% delivered within 6 hours of induction, and 10.1% had not delivered within 48 h. This also reflects in the frequency of CS due to failed induction of 4.3%. Correspondingly mean time from induction to delivery for the women induced with oral misoprostol was almost double; 49.8 h, 1.2% delivered within 6 h of induction, and 51.1% had not delivered within 48 h. CS due to failed induction was 11% in women induced with oral misoprostol.

**Table 3:** Secondary outcomes

	Vaginal Insert	Oral tablets	RR	95% CI
Caesarean section, n (%)	37 (31.9)	52 (30.2)	1.06	*0.70–1.5*
CS failed induction, n (%)	5 (4.3)	19 (11)	0.39	0.15–1.02
CS threatening asphyxia, n (%)	13 (11.2)	16 (9.3)	1.2	0.60–2.4
Tocolysis, n (%)	10 (8.6)	0	31	1.8–525
Scalp-pH, n (%)	49 (42)	42 (24.4)	1.69	1.20–2.38
Time to vaginal delivery (h), mean	23.7	46.2		*p* < 0.0001
Quick delivery^a^, n (%)	8 (6.9)	2 (1.2)	6.0	1.3–28
Slow delivery^b^, n (%)	12 (10.3)	88 (51.1)	0.20	0.12–0.35
Oxcytocin stimulation, n (%)	52 (44.8)	130 (75.6)	0.59	0.48–0.74
Balloon catheter, n (%)	7 (6.0)	37 (21.5)	0.28	0.13–0.61
Artificial rupture of membranes, n (%)	51 (44.0)	123 (71.5)	0.61	0.49–0.77
Fever^c^, n (%)	2 (1.7)	12 (7.0)	0.25	0.06–1.08
Epidural, n (%)	52 (44.8)	134 (77.9)	0.58	0.46–0.71
Prolonged rupture of membranes^d^, n (%)	4 (3.4)	17 (9.9)	0.35	0.12–1.01
Postpartum haemorrhage^e^, n (%)	9 (7.8)	18 (10.5)	0.74	0.35–1.59
Sphincter rupture, n (%)	6 (5.2)	6 (3.5)	1.62	0.54–4.9
Instrumental delivery, n (%)	19 (16.4)	31 (18.0)	0.91	0.54–1.5

^a^< 6 h, ^b^> 48 h, ^c^> 38.5 °C, ^d^. > 24 h, ^e^> 1 L

Consistent with these findings is the use of secondary methods to induce labour (double balloon catheter) which was 6% for vaginal misoprostol insert and 16.9% for oral misoprostol (RR 0.28; CI 0.13–0.61). Oxytocin stimulation was used in 44.8 and 75.6% respectively (RR 0.59; CI 0.48–0.74), and artificial rupture of membranes was used in 44.0% vs 71.5% of the women in the two groups (RR 0.61; CI 0.49–0.77). The three additional methods of induction were significantly different in the two groups.

We found a tendency towards more complications with a slower delivery, although not statistically significant. Prolonged rupture of membranes was 3.4% vs 9.9% (RR 0.35; CI 0.12–1.01) and Pyrexia (rectal temperature above 38.5 °C) was reported in 1.7% vs 7.0% of cases (RR 0.25; CI 0.06–1.08).

The use of epidural was significantly higher in the oral misoprostol group; 44.8% vs. 77.9% (RR 0.58; CI 0.46–0.71).

Also, the frequency of tachysystole, the use of tocolysis and additional fetal monitoring with scalp-pH, was significantly higher for the women induced with vaginal misoprostol (Table 3).

Rates of postpartum hemorrhage, instrumental delivery and sphincter rupture were similar in the two groups.

The results for a subgroup analysis for women with a body-mass-index above 30 showed a tendency towards a slower induction and higher risk of failed induction in both groups (Additional file 1: Table S3). The same analysis for immature cervical conditions, set at a Bishop score below four, did not show the same consistency in the numbers, although there seemed to be a higher risk of CS.

## Discussion

In this prospective cohort study, we assessed the safety and efficacy of misoprostol for induction of labour in nulliparous women, with two different routes of administration; 25 mcg oral tablets and 200 mcg slow release vaginal-insert in a total of 288 women. We found no significant difference in women achieving vaginal delivery. For safety aspects, this study found a significantly increased risk of tachysystole for the vaginal administration route; 28.4% compared with 2.3% (RR 12.2; CI 4.5–34). However, there were no events of serious neonatal asphyxia in this group. The time from induction to delivery was significantly shorter for the vaginal administration route, where more than half the women had delivered within 24 h, compared with 16.8% in the oral administration group, leading to smaller risk of prolonged rupture of membranes and pyrexia, although not significant. The efficacy of vaginal insert of misoprostol also led to a significantly decreased use of additional augmentation methods such as artificial rupture of membranes, balloon-catheter and use of oxytocin. The number of failed inductions leading to CS was also decreased. We found a tendency of increased risk of developing pyrexia in the women who received oral tablets. Nevertheless, the difference was not statistically significant probably due to the size of our material. Pyrexia has previously been reported as a side effect to misoprostol when used in higher doses for prevention of post-partum hemorrhage [13–15], but it may also be attributed to the increased risk of prolonged rupture of membranes that was observed in this study.

The present study was subject to methodological limitations as it was neither randomised nor blinded and set in two different hospitals. However, apart from inclusion of women with gestational diabetes the two populations were comparable and a subanalysis excluding the women with gestational diabetes did not change the results (Additional file 1: Table S1 and S2). Another limitation of the study was the in- vs out-patient regimen for the induction, where tachysystole could be thoroughly observed in the in-patient group (vaginal insert), and may have been underestimated in the out-patient setting (oral tablets).

Further limitations of the present study were a lack of data on all neonatal morbidity (i.e. admission to a neonatal department, proven bacterial infection, cooling and seizures etc.) due to restricted permissions for accessing the children’s medical records. Therefore, neonatal asphyxia is the only reported item on neonatal morbidity.

Since the study was not blinded the healthcare professionals might have contributed to inadvertent bias. Healthcare professionals were aware of the risk of tachysystole prior to the present trial and may have been prone to act outside of the guidelines for the regimen. The midwives may have been affected by this in choosing induction method (artificial rupture of membranes or medical induction), and this could subsequently be reflected in the slightly lower Bishop score for the women induced with vaginal misoprostol had.

Unfortunately, the desired number of women included in this study was not reached due to logistical matters resulting in a weaker statistical power.

No previous studies have compared misoprostol vaginal slow-release insert with oral misoprostol. The present study included only nulliparous women at term with intact membranes leading to two relatively homogenous study-groups.

Women’s experiences on a fast vs a slow delivery have not previously been investigated, which will be reported for the women included in this study in a separate publication.

Previous studies on nulliparous women with doses of oral misoprostol between 25 and 50 mcg found that delivery within 24 h occurred in 15 and 36% respectively; which is similar to our results [16, 17]. Rates of CS were reported as 13.6–32% in the same studies.

The misoprostol slow-release vaginal insert has been assessed in five previous studies, also including multiparous women, that found consistent results compared with ours. Time from induction to delivery has been reported between 14.5–26.6 h and rates for CS was 7.5–40.1 [17–23]. Frequency of hyperstimulation was reported between 4.0 and 48.1%, leading to a higher CS rate and negative effects on neonatal outcomes. In the present study, the women induced with vaginal insert were hospitalised from the time of induction and therefore monitored thoroughly. When tachysystole occurred, health care professionals were able to take relevant measurements and we did not observe any adverse outcomes in CS rates and serious neonatal asphyxia.

With the observed risk of tachysystole for vaginal insert, it is of essence that the woman and healthcare professionals are informed of the risks and induction is commenced in an in-patient setting to ensure patient safety, where there are means to take appropriate action if tachysystole occurs. Vaginal insert with misoprostol has some advantages compared with oral misoprostol tablets and there may be some individuals who could benefit from this method of induction, for instance where induction of labour is expected to be particularly difficult and/or prolonged. In a subgroup analysis on women with BMI > 30 or immature cervical conditions (Bishop score ≤ 4), the results were consistent with the whole groups, and these subgroups of women does not seem to benefit from one method of induction over the other (Additional file 1: Table S3).

A Cochrane analysis in 2014 stated, “Any proposed dose regimen includes a trade-off between rapid birth and uterine hyperstimulation” [5]. This seems evident in the present study where not only the risk of tachysystole is a matter of safety, but also the length of an induction and consequently the risk of prolonged rupture of membranes, antibiotics, pyrexia and failure of induction is a matter of safety both for the woman and her child.

Besides safety being the major concern for labour induction, there are also a number of practical implications to consider in choosing a method of induction. In this study, oral misoprostol appeared to be safe in an outpatient setting, whereas the vaginal insert only could be recommended to use when the patient is hospitalised and monitored thoroughly. From an economical perspective, induction of labour is costly, in both medication/devise expenses and staff-time. The average price for induction of labour varies between countries and the local sett-up at the hospital, and have therefore not been calculated in the present study. However, the additional number of medications and devises (i.e. oxytocin stimulation, double balloon catheter, epidural and antibiotics) were significantly higher the oral misoprostol group of women. The average staff time spent on labour inductions is seemingly higher for the women induced with oral misoprostol, although the majority of the women in this group was induced in an outpatient setting and the time from induction to delivery is not a reasonable estimate for the staff-time spent on the induction.

## Conclusion

Compared with a regimen with oral misoprostol we found that induction with vaginal slow-release misoprostol leads to faster delivery with increased risk of tachysystole but with similar perinatal outcome and rates of CS. Low-dose oral misoprostol appears to be safe, but leads to an increased use of secondary methods and a tendency of more intrapartum pyrexia.

### Tweetable abstract

Labour induction with misoprostol: a cohort study – safe and slow (oral tablets) or quick and efficient (vaginal insert)?

## Supplementary information

**Additional file 1: Table S1.** Subgroup analysis of patient characteristics excluding GDM. **Table S2.** Primary and secondary outcomes excluding GDM. **Table S3.** Subgroup analysis of secondary outcomes on BMI > 30 and Bishop score ≤ 4

## Data Availability

All data analysed during this study are included in this published article. All generated raw data used for the analysis was deleted after completion of analysis as advised from the data protection authorities.

## Abbreviations

CI: Confidence Interval
CS: Cesarean Section
CTG: Cardiotocography
GDM: Gestational Diabetes Mellitus
RR: Relative Risk
