# Supplementary material for: Induction of labour in nulliparous women- quick or slow: a cohort study comparing slow-release vaginal insert with low-dose misoprostol oral tablets
Source: BMC Pregnancy Childbirth. 2020 Feb 7;20:79. doi: 10.1186/s12884-020-2770-0 (PMC7006088; doi:10.1186/s12884-020-2770-0)
Supplement: Supplementary file 1 — Additional file 1: Table S1. Subgroup analysis of patient characteristics excluding GDM. Table S2. Primary and secondary outcomes excluding GDM. Table S3. Subgroup analysis of secondary outcomes on BMI > 30 and Bishop score ≤ 4 [file 12884_2020_2770_MOESM1_ESM.docx]

| Supplementary table 1: Subgroup analysis of patient characteristics excluding GDM | | | |
| --- | --- | --- | --- |
| **Induction method** | **Vaginal insert (n=115)** | **Oral tablets excluding GDM (n=135)** | **5% p-value** |
| **Age** (years), mean (SD) | 27.8 (5.0) | 27.3 (5.0) | 0.43 |
| **BMI** (kg/m^2^) mean (SD) | 27.7 (8.4) | 29.3 (7.9) | 0.12 |
| **Cigarette use,** n (%) | 20 (17) | 20 (14.8) | 0.58 |
| **Pre-existing medical conditions,** n (%) | 12 (10.3) | 18 (13.3) | 0.48 |
| **Pre-existing psychiatrical conditions,** n (%) | 12 (10.3) | 15 (11.1) | 0.86 |
| **Pregnancy-related medical conditions,** n (%)  *Preeclampsia*  *Hypertension*  *Intrahepatic cholestasis*  *Others* | 31 (26.7)  *16 (13.8)*  *2 (1.7)*  *3 (2.6)*  *9 (7.8)* | 42 (31.1)  *22 (16.3)*  *9 (6.7)*  *5 (3.7)*  *6 (4.4)* | 0.47  0.60  0.06  0.62  0.26 |
| **Indication for induction,** n (%)  *Medical/Obstetrical Post-dates^1^*  *Other* | 45 (39)  57 (49)  13 (11.2) | 78 (57.8)  54 (40.0)  3 (2.2) | 0.003  0.13  0.003 |
| **Bishop score,** mean (SD) | 3.2 (1.5) | 4.3 (2.1) | <0.001 |
| **Gestational age at delivery** (w+d), mean (SD) | 40+5 (1+3) | 40+5 (1+3) | 1 |
| **Birthweight** (g), mean (SD) | 3636 (511) | 3640 (543) | 0.95 |
| *1. Gestational age above 41+3* | | | |

| Supplementary table 2: Primary and secondary outcomes excluding GDM | | | | |
| --- | --- | --- | --- | --- |
| **Induction method** | **Vaginal insert**  **n=115** | **Oral tablets excluding GDM n=135** | **RR** | **95%CI** |
| **Caesarean section,** n (%) | 37 (31.9) | 40 (29.6) | 1.07 | 0.7-1.6 |
| **Tachysystole,** n (%) | 33 (28.4) | 3 (2.2) | 12.8 | 4.0-40 |
| **Tachysystole with category III fetal heart rate patterns,** n (%) | 13 (11.2) | 2 (1.5) | 7.6 | 1.7-33 |
| **Delivery within 24 h,** n (%) | 65 (56.0) | 20 (14.8) | 3.8 | 2.4-5.8 |
| **Neonatal Asphyxia,** n (%) | 0 | 2 (1.5) | 0.2 | 0.1-4.9 |
| **CS failed induction,** n (%) | 5 (4.3) | 13 (9.6) | 0.45 | 0.16-1.21 |
| **CS threatening asphyxia,** n (%) | 13 (11.2) | 13 (9.6) | 1.16 | 0.56-2.41 |
| **Tocolysis,** n (%) | 10 (8.6) | 0 | 24 | 1.45-412 |
| **Scalp-pH,** n (%) | 49 (42) | 31 (23.0) | 1.84 | 1.26-2.68 |
| **Time to vaginal delivery** (h), mean | 23.7 | 48.5 |  | p<0.0001 |
| **Quick delivery**^3^**,** n (%) | 8 (6.9) | 1 (0.7) | 9.31 | 1.18-73.3 |
| **Slow delivery**^4^**,** n (%) | 12 (10.3) | 61 (45.2) | 0.23 | 0.13-0.40 |
| **Oxcytocin stimulation,** n (%) | 52 (44.8) | 99 (73.3) | 0.61 | 0.48-0.77 |
| **Balloon catheter,** n (%) | 7 (6.0) | 30 (22.2) | 0.27 | 0.12-0.60 |
| **Artificial rupture of membranes,** n (%) | 51 (44.0) | 96 (71.1) | 0.65 | 0.51-0.81 |
| **Fever**^5^**,** n (%) | 2 (1.7) | 10 (7.4) | 0.23 | 0.05-1.04 |
| **Epidural,** n (%) | 52 (44.8) | 102 (75.6) | 0.59 | 0.47-0.74 |
| **Prolonged rupture of membranes**^6^**,** n (%) | 4 (3.4) | 14 (10.4) | 0.33 | 0.11-0.98 |
| **Postpartum haemorrhage^7^,** n (%) | 9 (7.8) | 11 (8.1) | 0.95 | 0.48-2.22 |
| **Sphincter rupture,** n (%) | 6 (5.2) | 6 (4.4) | 1.16 | 0.39-3.51 |
| **Instrumental delivery,** n (%) | 19 (16.4) | 23 (17.0) | 0.96 | 0.55-1.67 |

| Supplementary table 3: Subgroup analysis of secondary outcomes on BMI > 30 and Bishop score ≤4 | | | | |
| --- | --- | --- | --- | --- |
| **Induction method** | **Vaginal Insert** | **Oral tablets** | **RR** | **95% CI** |
| **Caesarean section**, n (%)  *BMI >30*  *Bishop ≤4* | 37 (31.9)  *13 (38.3)*  *30 (33.0)* | 52 (30.2)  *18 (30.5)*  *37 (34.6)* | 1.06  *1.25*  *0.95* | *0.7-1.5*  *0.71-2.23*  *0.64-1.41* |
| **CS failed induction,** n (%)  *BMI >30*  *Bishop ≤4* | 5 (4.3)  *3 (8.8)*  *5 (5.5)* | 19 (11)  *9 (15.3)*  *15 (14.0)* | 0.39  *0.58*  *0.39* | 0.15-1.02  *0.17-1.99*  *0.15-1.04* |
| **Time to vaginal delivery** (h), mean  *BMI >30*  *Bishop ≤4* | 23.7  *31.5*  *24.5* | 46.2  *52.8*  *51.3* |  | p<0.0001  *p=0.0004*  *p<0.0001* |
| **Quick delivery**^1^**,** n (%)  *BMI >30*  *Bishop ≤4* | 8 (6.9)  *1 (2.9)*  *5 (5.5)* | 2 (1.2)  *0*  *1 (1.0)* | 6.0  *5.1*  *5.9* | 1.3-28  *0.21-123*  *0.70-49* |
| **Slow delivery**^2^**,** n (%)  *BMI >30*  *Bishop ≤4* | 12 (10.3)  *6 (17.6)*  *11 (12.1)* | 88 (51.1)  *33 (56)*  *62 (58)* | 0.20  *0.32*  *0.21* | 0.12-0.35  *0.15-0.68*  *0.12-0.37* |
| **Balloon catheter,** n (%)  *BMI >30*  *Bishop ≤4* | 7 (6.0)  *5 (14.7)*  *6 (6.6)* | 37 (21.5)  *16 (27.1)*  *29 (27.1)* | 0.28  *0.54*  *0.24* | 0.13-0.61  *0.22-1.35*  *0.11-0.56* |
| **Fever**^3^**,** n (%)  *BMI >30*  *Bishop ≤4* | 2 (1.7)  *2 (5.9)*  *2 (2.2)* | 12 (7.0)  *6 (10.2)*  *10 (9.3)* | 0.25  *0.58*  *0.24* | 0.06-1.08  *0.12-2.71*  *0.05-1.05* |
| **Prolonged rupture of membranes**^4^**,** n (%)  *BMI >30*  *Bishop ≤4* | 4 (3.4)  *4 (11.8)*  *2 (2.2)* | 17 (9.9)  *9 (15.3)*  *14 (13.1)* | 0.35  *0.77*  *0.17* | 0.12-1.01  *0.26-2.32*  *0.04-0.75* |
| *1. <6h; 2. >48h; 3. > 38.5ºC; 4. >24hours* | | | | |
